# Immunogenic *Eimeria tenella* Glycosylphosphatidylinositol-Anchored Surface Antigens (SAGs) Induce Inflammatory Responses in Avian Macrophages

**DOI:** 10.1371/journal.pone.0025233

**Published:** 2011-09-28

**Authors:** Yock-Ping Chow, Kiew-Lian Wan, Damer P. Blake, Fiona Tomley, Sheila Nathan

**Affiliations:** 1 School of Biosciences and Biotechnology, Faculty of Science and Technology, Universiti Kebangsaan Malaysia, Bangi, Selangor D.E., Malaysia; 2 Malaysia Genome Institute, Kajang, Selangor D.E., Malaysia; 3 Pathology and Infectious Diseases, Royal Veterinary College, University of London, North Mymms, United Kingdom; Universidade Federal de Minas Gerais, Brazil

## Abstract

**Background:**

At least 19 glycosylphosphatidylinositol (GPI)-anchored surface antigens (SAGs) are expressed specifically by second-generation merozoites of *Eimeria tenella*, but the ability of these proteins to stimulate immune responses in the chicken is unknown.

**Methodology/Principal Findings:**

Ten SAGs, belonging to two previously defined multigene families (A and B), were expressed as soluble recombinant (r) fusion proteins in *E. coli*. Chicken macrophages were treated with purified rSAGs and changes in macrophage nitrite production, and in mRNA expression profiles of inducible nitric oxide synthase (iNOS) and of a panel of cytokines were measured. Treatment with rSAGs 4, 5, and 12 induced high levels of macrophage nitric oxide production and IL-1β mRNA transcription that may contribute to the inflammatory response observed during *E. tenella* infection. Concomitantly, treatment with rSAGs 4, 5 and 12 suppressed the expression of IL-12 and IFN-γ and elevated that of IL-10, suggesting that during infection these molecules may specifically impair the development of cellular mediated immunity.

**Conclusions/Significance:**

In summary, some *E. tenella* SAGs appear to differentially modulate chicken innate and humoral immune responses and those derived from multigene family A (especially rSAG 12) may be more strongly linked with *E. tenella* pathogenicity associated with the endogenous second generation stages.

## Introduction

*Eimeria tenella* is one of the most pathogenic *Eimeria* spp. that cause avian coccidiosis and inflicts great economic losses on the world poultry industry [1]. Haemorrhage and extensive destruction of caecal tissues are the major pathological manifestations of *E. tenella* infection caused predominantly by rapid growth of second-generation schizonts within crypt epithelial cells that migrate deep into the lamina propria, and the rupture of these schizont-infected cells to release second-generation merozoites [2], [3]. Merozoites are highly immunogenic [4], [5] and contain proteins capable of inducing immune responses in the host [6]. Glycosylphosphatidylinositol (GPI)-anchored surface antigens (SAGs) of *E. tenella* are among the major surface molecules of the parasite and many of these are expressed during the development of second generation merozoites [7] making them good targets for host innate and adaptive immune responses. GPI-linked antigens are also expressed on the surfaces of other apicomplexan parasites such as *Plasmodium falciparum*, *Sarcocystis neurona* and *Toxoplasma gondii*. Some of these surface antigens are known to elicit strong immune responses and have been associated with a variety of functions in host cell invasion, immune evasion, and pathogenicity [8]–[10]. Understanding the role that *E. tenella* SAGs have with respect to the chicken immune response should provide insights into control of coccidiosis.

During an *E. tenella* infection of chickens, macrophages massively infiltrate into the chicken caecal lamina propria on day-1 post-infection and secrete large amounts of cytokines [11]–[13]. In addition, activated macrophages are attracted to the inflammation site resulting in an increased severity of infection [11], [14], [15]. Macrophages are key immunocytes of the host innate immune response and produce cytokines which essentially shape the type of acquired immune response and outcome of an infection upon confronting a pathogen [16]. Generally, cytokines such as IL-12, IFN-γ, IL-1β and TNF-α promote the development of cellular mediated immunity against intracellular infections including coccidiosis [1]. This group of cytokines is associated with inflammatory responses, whilst cytokines such as IL-10 favour the development of humoral mediated immunity and are implicated in anti-inflammatory responses [16]. In addition, nitric oxide (NO) regulated by inducible nitric oxide synthase (iNOS) is an important molecule produced upon macrophage activation. NO acts directly as an effector molecule against invading pathogens but can also be cytotoxic to the host if it is over produced [17], [18]. *E. tenella* infection triggers both humoral and cellular mediated immune responses in the chicken [1], although antibody mediated immunity appears to play a minor role in protecting chickens during natural *Eimeria* infections [19]. Nevertheless, several studies have shown that antibodies against *Eimeria* proteins, either administered parenterally or transferred to the hatching via the yolk following maternal immunisation, can partially protect against coccidiosis [1]. A cocktail of immunogens capable of triggering high-titer antibody responses could potentially provide complete protection against *Eimeria* infection [20].

Several studies have been conducted to understand the interplay between *E. tenella* infection and the chicken immune response; however the role(s) of parasite surface antigens in mediating chicken innate or adaptive immune responses is yet to be understood. Bioinformatics and cDNA analysis identified at least 19 SAGs that are specifically expressed in *E. tenella* second-generation merozoites which can be grouped into two multi-gene families, A and B [7]. In this study, five SAGs were chosen at random from each of these families to elucidate their potential role as immune effectors (Family A: SAGs 2, 3, 4, 5, 12; Family B: SAGs 15, 16, 18, 19, 23). Recombinant *E. tenella* SAGs (rSAGs) were produced in *Escherichia coli* as soluble thioredoxin (Trx) fusion proteins, purified individual rSAGs were incubated with chicken macrophages and macrophage NO production, as well as changes in iNOS, IL-1β, IL-12, IFN-γ and IL-10 mRNA transcription levels, were measured. The relevance of rSAGs to the chicken humoral immune response was also determined through their reactivity with antibodies from infected and uninfected chickens. Overall, our findings provide new sights on the roles of *E. tenella* surface antigens in modulating chicken immune responses.

## Results

### Expression and purification of rSAGs

Ten (SAGs 2, 3, 4, 5, 12, 15, 16, 18, 19 and 23) *E. tenella* SAG cDNAs were successfully amplified without the hydrophobic signal peptide and GPI-anchored region, cloned into pTZ57R/T vector and subcloned into the pET32b(+) for expression in *E. coli* Rosetta gami (DE3). SDS-PAGE and western blotting demonstrated successful rSAG expression as soluble Trx fusion proteins following low temperature incubation and induction with 0.1 mM IPTG (Figure S1). The expressed rSAGs fused to Trx were within the predicted size range (41–43 kDa). The expressed soluble recombinant proteins were purified by immobilised nickel affinity chromatography under native conditions. These purified rSAGs (Figure S1) were then utilised to stimulate macrophages and also screened against convalescent chicken sera to confirm their immunogenicity.

### Effect of rSAGs on macrophage NO production

**Figure 1:**
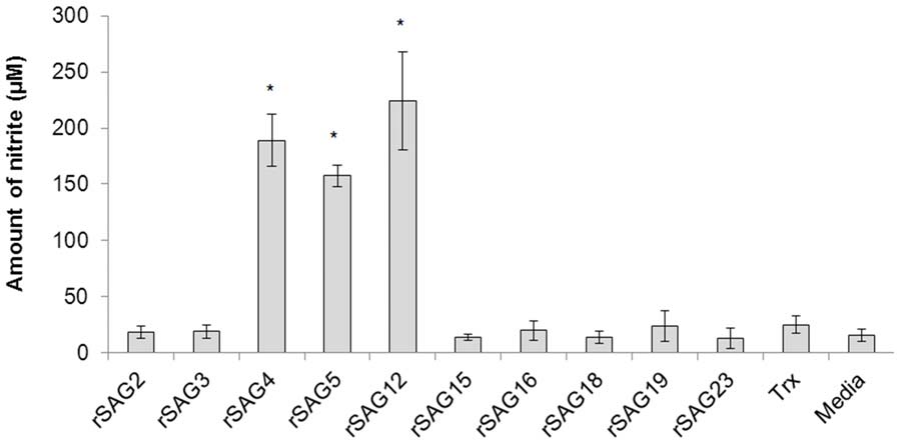
Effect of rSAGs on the nitrite production by chicken macrophage HTC cells. Chicken macrophage HTC cells were exposed to 100 µg/mL of each rSAG for 24 h and the levels of nitrite was measured by Griess assay. The results are expressed as mean±SD of three independent experiments. *, P<0.05 was considered significant compared to untreated cells as analysed by unpaired Student's t-test.

**Figure 2:**
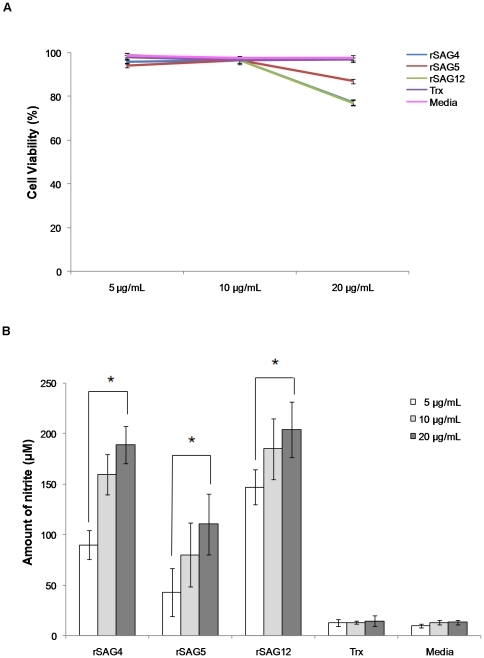
Effects of various concentration of rSAGs 4, 5, 12 and Trx on the viability and nitrite production of chicken macrophage HTC cells. Chicken macrophage HTC cells were exposed to different concentrations (5, 10 and 20 µg/mL) of rSAGs 4, 5 and 12 and Trx for 24 h. (A) Macrophages viability was determined by trypan blue exclusion assay. The viability of macrophages became affected at 20 µg/mL of each rSAG. The results are expressed as mean±SD of three independent experiments. (B) The nitrite production by macrophages was determined by Griess assay. Nitrite production increased from treatment with 5 µg/mL to 20 µg/mL rSAG. *, P<0.05 was considered significant as analysed by unpaired Student's t-test.

To assess the ability of rSAGs 2, 3, 4, 5, 12, 15, 16, 18, 19 and 23 to induce production of macrophage NO (measured by nitrite production), cells were treated with 100 µg/mL of each rSAG for 24 h and supernatants were harvested to determine nitrite levels. Our results demonstrated that the viability of macrophages treated with rSAGs 4, 5 and 12 was reduced (data not shown) while a concomitant increase in levels of NO production (Figure 1) was also observed. On the other hand, cells treated with other seven rSAGs, Trx or media alone presented low levels of NO. These cells, nevertheless, remained viable (>95%) when examined by the trypan blue exclusion assay (data not shown). Therefore, the inability of the seven rSAGs to induce NO production was not due to cell death. To reduce the toxicity of the selected recombinant SAGs on the macrophage cells, concentrations of rSAGs 4, 5 and 12 were then reduced to 5, 10 or 20 µg/mL (Figure 2A). NO production clearly increased in a dose-dependent manner in cells treated with 5, 10 or 20 µg/mL (p<0.05) (Figure 2B), however cell viability was affected by treatment with 20 µg/mL of all three SAGs tested. Therefore, for all subsequent studies, rSAGS 4, 5 and 12 at a concentration of 10 µg/mL were used.

The LPS-inactivating agent polymyxin B sulphate (PMB) was added to cells treated with stimulants to rule out the possibility of NO production due to *E. coli* endotoxin contamination. The addition of PMB did not affect rSAG or *E. tenella* merozoite crude lysate-induced NO production (Figure S2). However, PMB was shown to significantly reduce NO production in cells treated with 1 µg/mL LPS (p<0.001), hence verifying that the effect was specific to the rSAGs. The fusion partner Trx failed to trigger macrophage NO production. In addition, it is also noteworthy that all the stimulants used in this study contained less than 1 ng/mL of endotoxins as determined by the *Limulus* Amebocyte assay.

### Effects of stimulants on macrophage cytokine and iNOS production

**Figure 3:**
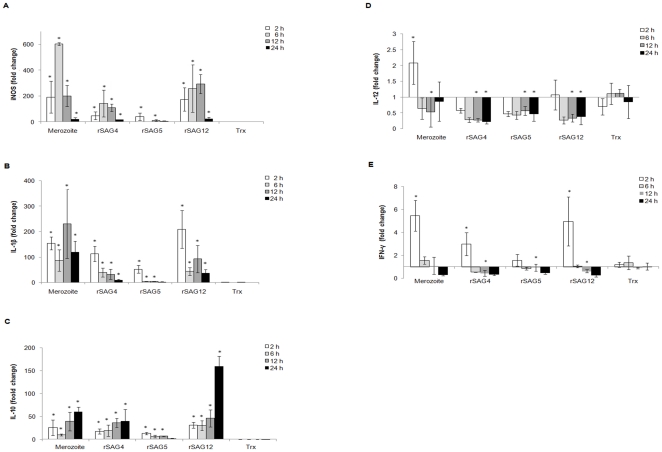
Effects of *Eimeria tenella* merozoite crude lysate, rSAGs 4, 5 and 12 and Trx on chicken macrophage cytokines and iNOS expression. Chicken HTC macrophages were treated with 10 µg/mL of *Eimeria tenella* merozoite crude lysate, rSAGs 4, 5 and 12 and Trx for 2, 6, 12 and 24 h. The mRNA transcriptional levels of (A) iNOS; (B) IL-1β; (C) IL-10; (D) IL-12 and (E) IFN-γ were determined by qPCR. Bars represent fold change relative to untreated cells from three independent experiments. *, P<0.05 was considered significant compared to cells treated with Trx as analysed by unpaired Student's t-test.

In order to determine the influence of rSAGs 4, 5, 12, Trx and merozoite crude lysate on cytokine and iNOS expression, treated and untreated macrophages were harvested at 2, 6, 12 and 24 h post-stimulation to measure changes in mRNA transcription levels. As shown in Figure 3A, iNOS mRNA transcription was significantly up regulated as early as 2 h post-stimulation following rSAG treatment, with rSAG12 the most potent stimulator followed by rSAG4 and rSAG5. A similar profile was observed upon treatment with *E. tenella* merozoite crude lysate. We suggest therefore that the production of high levels of nitrite (as shown in Figure 2) was a result of up-regulation of iNOS expression.

As shown in Figure 3B, all stimulants significantly increased levels of IL-1β mRNA transcripts in avian macrophages compared to cells treated with Trx over 24 h (P<0.05). Merozoite crude lysate appeared to be the most potent stimulant (88–231 fold change) followed by rSAG12 (37–209 fold), rSAG4 (9–113 fold) and rSAG5 (5–52 fold) over 24 h. Furthermore, as depicted in Figure 3C, the stimulants induced a moderate increase in IL-10 mRNA transcription over 24 h when using rSAG12 (30–160 fold), rSAG4 (18–41 fold), rSAG5 (2–13 fold) and merozoite crude lysate (10–61 fold). Meanwhile, IL-12 mRNA transcription was significantly down regulated at 12 and 24 h post-stimulation by all three rSAGs (Figure 3D). For IFN-γ, mRNA transcription levels were briefly elevated at a low level (3–5 fold) but later down regulated significantly at 12 h post-stimulation by each rSAG (Figure 3E).

Overall, the alteration of macrophage cytokine and iNOS mRNA transcript levels was found to be specific to the three rSAGs. rSAGs 4, 5 and 12 appear to be able to modulate macrophage cytokine and iNOS transcript production at levels comparable to stimulation with *E. tenella* merozoite crude lysate. rSAG12 was the most potent immunomodulator, followed by rSAG4 and rSAG5.

### Reactivity of chicken antibodies towards rSAGs

**Figure 4:**
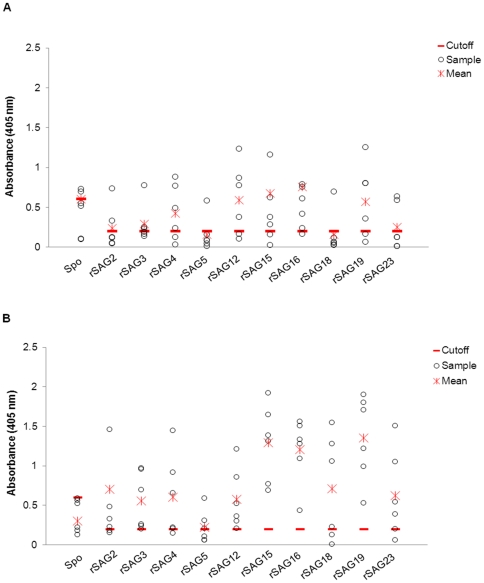
Reactivity of day 8 and day 14 post-infected chickens sera with crude sporulated oocyst lysate and rSAGs in ELISA. Crude sporulated oocyst lysate and rSAGs were screened against sera collected from individual *Eimeria tenella* infected chickens on (A) day 8 (n = 6) and (B) day 14 (n = 6) post-infection. Cut off values are indicated by the horizontal line, mean values are indicated by asterisk symbol.

rSAGs and Trx were screened against infected and uninfected chicken sera to determine their immunogenicity. The absorbance reading specific for each rSAG was obtained after subtracting the reading for the Trx fusion partner control. All of the selected rSAGs were detected by sera collected from infected chickens on day 8 (Figure 4A) and day 14 (Figure 4B) post-infection but not on day 7. Concomitantly, sera samples from uninfected birds obtained at similar time points did not react with the rSAGs. Comparison between infected chickens demonstrated 33–100% reactivity, which was comparable to the crude *E. tenella* sporulated oocyst lysate used as the positive control (50–83% positive; Table S2). The mean absorbance reading (Figure 4A, 4B) indicated that antibody levels against rSAGs increased from day 8 to day 14 post-infection. rSAGs 15, 16 and 19 appear to be more immunogenic with higher antibody reactivity, whereas rSAG5 was the least immunogenic. A screen for IgM and IgA antibodies towards the rSAGs was consistently negative (data not shown).

## Discussion

A number of studies have demonstrated that *E. tenella* is capable of eliciting both innate and acquired immune responses in chickens [1]; however the molecules responsible for triggering these immune responses remain largely unknown. *In silico* analyses of expressed sequence tags of *E. tenella* sporozoites and second-generation merozoites identified at least 19 merozoite-specific SAGs that were classified into two multigene families, A and B based on the positions of six conserved cysteines [7]. Armed with the evidence that *E. tenella* merozoites are highly immunogenic and that multiple SAGs are expressed on their surfaces [5], [7], the present study focused on characterising the roles of a subset of merozoite stage-specific SAGs from families A and B in inducing chicken innate and humoral immune responses as a step towards the development of improved vaccines and/or diagnostic tools.

Resting macrophages lack the iNOS required to synthesise large amounts of NO and expression of iNOS is initiated by a variety of stimuli including pathogens or pathogen associated molecules [17], [21]. In spite of its role in eliminating the infectious agent, excessive production of NO can also be detrimental to the host [15], [22],[23]. Our studies have shown that incubation of avian HTC macrophages with rSAGs 4, 5 and 12 results in the production of high amounts of NO, apparently regulated by the expression of iNOS, with comparable levels observed when using *E. tenella* merozoite crude lysate. Conversely, no increase in macrophage NO production was observed when using other rSAGs as stimulatory factors. The levels of iNOS induced by merozoite crude lysates, rSAGs 4, 5 and 12 are concomitant with those observed in vivo [24], [25]. We therefore postulate that SAGs 4, 5 and 12 have the potential to be detrimental to the host given that NO can induce haemorrhage, a major pathological manifestation of *E. tenella* infected caeca [3]. Previous reports have also demonstrated high levels of iNOS following infection by *E. tenella* but not *Eimeria acervulina* and *Eimeria maxima* [25], supporting the role of NO in the inflammatory and haemorrhagic pathology more frequently observed in *E. tenella* induced caecal coccidiosis. SAG gene repertoires and expression profiles are yet to be determined for *E. acervulina* and *E. maxima*, causes of malabsorptive coccidiosis, and may be distinct from that observed for *E. tenella*. The specific stimulation of NO production by the SAGs investigated here may assist *E. tenella* merozoite invasion in the caeca in a similar manner to that described for the excretory-secretory antigens of *Toxocara canis* [26].

Overproduction of the proinflammatory cytokine IL-1β can be detrimental to the host. For example, in studies with shigellosis blocking IL-1β activity using an IL-1 receptor antagonist in a rabbit-ligated loop model decreased bacterial invasion, implicating IL-1-dependent inflammation as important for bacterial invasion [27]. Furthermore, early inflammatory responses in a murine lung model of infection were decreased in IL-1β knockout mice although bacterial clearance was similar when compared with wild-type mice [28]. For caecal coccidiosis, elevated expression of IL-1β was previously reported during *E. tenella* infection [13], [24], [25]. Our results demonstrate that merozoite crude lysates induce high levels of IL-1β mRNA transcription over 24 h and for rSAGs 4, 5 and 12 stimulation was observed within 2 h. Thus, these proteins may contribute to the stimulation of the inflammatory immune responses associated with merozoite invasion and replication.

IL-12 and IFN-γ are key cytokines orchestrating the development of cellular mediated immunity and their expression has been shown to be down regulated by the induction of IL-10 [29]. IL-12 plays an essential role in protecting the host against parasitic disease and its abrogation increases host susceptibility. For instance, mice deficient for IL-12 fail to develop a protective immune response and succumb during acute *T. gondii* infection [30] and fail to control infection by *Leishmania major* [31], [32]. During *E. maxima* infection, genetically susceptible chickens express more IL-10 than resistant chickens. IL-10 may be induced by the parasite to counteract the protective effects of IFN-γ which limit growth of the parasite [33]. Moreover, previous studies have also demonstrated that IFN-γ limits replication of *Eimeria*. For example, *E. tenella* replication was significantly inhibited after treatment with recombinant chicken IFN-γ [34]. Chicken macrophages or fibroblasts treated with crude culture supernatants containing IFN-γ exhibit reduced intracellular development of *E. tenella* [12]. Our results demonstrate that *E. tenella* merozoite crude lysate and some rSAGs are capable of moderately upregulating IL-10 mRNA transcription and down regulating IL-12 and IFN-γ mRNA transcription over 24 h.

Taken together, our results demonstrate that the immunomodulatory effect of some *E. tenella* rSAGs towards the chicken innate immune response is similar to that of merozoite crude lysates. We postulate that the high levels of IL-1β and iNOS stimulated by rSAGs are associated with intense inflammation, damaging caecal epithelial cells and enhancing invasion by *E. tenella*. rSAG induction of IL-10 mRNA transcription is closely related to the down regulation of both IL-12 and IFN-γ. Interestingly, SAGs 4, 5, and 12 are derived from SAG family A. The relevance of clusters of genes whose functions correlate with *E. tenella* immunopathology remains to be investigated. Even though seven of the rSAGs investigated here failed to trigger significant macrophage NO production, some of the family A SAGs ability to immunomodulate the expression of IL-12, IFN-γ and IL-10 are of relevance to our understanding of their biological function(s) and their influence on the acquired immune response.

All ten *E. tenella* rSAGs we examined are immunogens and were recognised by sera collected from infected chickens as early as day 8 post-infection. Antibodies targeting rSAGs became more common by day 14 post-infection, consistent with the findings that antibody levels peak on week 2 post-infection [35], [36]. rSAGs were not detected by serum IgA antibodies, as previously reported [37]. The identification of SAGs 2, 4 and 12 as immunogens was previously reported from an immunoproteomic analysis of second-generation *E. tenella* merozoite proteins [18]. We postulate that SAG proteins could be among the molecules utilised by *E. tenella* to confuse the host immune system and improve parasite survival. The simultaneous expression of multiple SAG proteins [7] might misdirect the chicken immune response towards antibody production rather than the cellular mediated immune responses required to eliminate *E. tenella*, thus allowing the parasites to avoid the host first line defence mechanisms and multiply more easily. Our data also support the notion that the immunogenicity of a protein should not be the main criterion for selecting potential vaccine candidates. As we have demonstrated, SAGs appear to be capable of triggering chicken humoral antibody responses and modulating cytokine responses to the parasite's advantage.

In summary, this study adds to our understanding of the ability of *E. tenella* SAGs to induce immune responses in the chicken. Our findings demonstrate that *E. tenella* SAGs 2, 3, 4, 5, 12, 15, 16, 18, 19 and 23 are immunoreactive proteins capable of eliciting chicken humoral immune responses. SAGs 4, 5 and 12 also appear to be capable of impairing development of host cellular mediated immunity and may contribute towards the inflammatory responses observed in infected chicken caeca.

## Materials and Methods

### Ethics Statement

All animal experiments were approved by the Universiti Kebangsaan Malaysia Animal Ethics Committee (UKMAEC), Malaysia, under approval number FST/SBB/2010/SHEILA/24-AUGUST/320-AUGUST-2010-NOVEMBER-2010.

### PCR amplification and cloning

*E. tenella* SAG transcripts were amplified from a merozoite cDNA library [38] using sets of forward (F) and reverse (R) primers incorporating *Nco*1 ( CCATGG ) and *Xho*1 ( CTCGAG ) restriction enzyme sites (Table S1). PCR assays were performed using high fidelity *Taq* polymerase (Roche) with initial denaturation at 95°C for 5 min and cycled 35 times at 95°C for 1 min, 55°C for 1 min and 72°C for 1 min. The reaction was completed with a final extension at 72°C for 10 min. The purified PCR products were cloned into the pTZ57R/T cloning vector (Fermentas) and subsequently subcloned into the pET32b(+) expression vector (Novagen). Plasmids were extracted from the clones and subjected to DNA sequencing to confirm the sequence and orientation of the inserts. The pET32b(+)_SAG plasmids were then transformed into *E. coli* Rosetta gami (DE3) (Novagen) for expression.

### Protein expression and purification

*E. coli* Rosetta gami (DE3) carrying individual pET32b(+)_SAGs or pET32b(+) alone were subcultured into Luria Broth (LB) supplemented with ampicillin (50 µg/mL) and kanamycin (30 µg/mL) at 37°C. Upon reaching an optical density (OD) at 600 nm of 0.5, protein expression was induced with 0.1 mM isopropylthio-D-galactoside (IPTG) for 20 h at 20°C. The cells were pelleted by centrifugation at 6000× g for 10 min and lysed using the Bug Buster Reagent (Novagen). The expressed soluble recombinant SAGs (rSAGs) fused to Trx, or Trx alone, were obtained from supernatants after centrifugation at 12,000× g for 20 min at 4°C. The expressed proteins were mixed with 70 mM imidazole and purified under native conditions through nickel-nitrilotriacetic acid (Ni-NTA) sepharose columns (Amersham) on the Akta Purifier system (GE, Healthcare). The columns were equilibrated with loading buffer (50 mM NaH_2_PO_4_, 1 M NaCl, 70 mM imidazole, pH 7.4) and the bound proteins were eluted with eluting buffer (50 mM NaH_2_PO_4_, 1 M NaCl, 300 mM imidazole, pH 7.4) over a 20 column volume in a linear gradient at a flow rate of 1 mL/min. Fractions containing the recombinant protein were pooled, desalted and buffer exchanged (PBS pH 7.4). The expressed and purified rSAGs were quantified by bicinchoninic acid assay (Sigma), and evaluated by sodium dodecyl sulphate polyacrylamide gel electrophoresis (SDS-PAGE) and western blot. For macrophage assays, endotoxins were removed from the protein solutions via a polymyxin B agarose column (Detoxi gel, Pierce). All the tested rSAGs contained less than 0.5 EU/µg protein as determined by the *Limulus* ameabocyte lysate (LAL) assay (QCL-1000 test kit, BioWhittaker).

### SDS-PAGE and western blot analysis

The expressed and purified rSAGs were separated on 12% SDS-PAGE [39] and stained with Coomassie blue. The presence of rSAG was confirmed by western blot [40]. The separated proteins were transferred onto nitrocellulose membranes (Amersham) and blocked with 5% skim milk in PBS. The membranes were then washed with washing buffer (PBS-0.1% Tween 20) followed by incubation with mouse anti-Histidine monoclonal antibody (Novagen; 1∶5000) for 1 h at 37°C. The membranes were then washed with washing buffer and incubated with horseradish peroxidase (HRP)-conjugated rabbit anti-mouse IgG antibody (Promega; 1∶5000) for 1 h at 37°C. The membranes were washed again and signals were detected using the Super Signal Enhanced Chemiluminescence System (Pierce).

### Determination of NO production from stimulated macrophages

Chicken macrophage HTC cells were provided by Dr. Narayan Rath from USDA Agricultural Research Service, USA [41] and were cultured in RPMI-1640 supplemented with 10% heat inactivated fetal bovine serum, 10 mM HEPES, 25 mM sodium pyruvate and 2 mM L-glutamine at 37°C in humidified air containing 5% CO_2_. Approximately 2×10^6^ chicken macrophage HTC cells per mL were seeded into 12-well tissue culture plates and grown overnight. The non-adherent cells were removed by washing with warm HBSS and the adherent cells (∼1×10^6^ cells) were incubated with 1 mL of medium alone or the same volume of medium containing 5, 10, 20 or 100 µg/mL of each rSAG for 24 h. The culture supernatants were collected and subjected to Griess assay. As controls, cells were incubated in the presence or absence of polymyxin B sulphate (50 µg/mL) while LPS (1 µg/mL) was used as a positive control.

### Griess assay

The concentration of nitrite, an end product from NO generated by HTC macrophages, was determined by Griess assay. Briefly, 50 µL of the supernatants collected from treated or untreated cells was incubated with 50 µL Griess reagent (Sigma) at room temperature for 15 min in a 96-well, flat bottom plate. The absorbance at 520 nm was determined on a microplate reader (Tecan, USA). Nitrite content was extrapolated from the sodium nitrite standard curve.

### Measurement of cytokine and iNOS mRNA transcripts from stimulated macrophages

Chicken macrophage HTC cells were seeded into 12-well plates at a density of ∼2×10^6^ cells/well. The cells were then treated with 10 µg/mL of rSAGs, Trx and merozoite crude lysates for 2, 6, 12 and 24 h. Total RNA was extracted from treated and untreated HTC cells using TRI Reagent according to manufacturer's instructions and treated with DNAse1 (Ambion). A total of 2 µg total RNA was reversed transcribed into cDNA in a total volume of 20 µL using Affinity Multitemp (Stratagene) and subjected to quantitative polymerase chain reaction (qPCR) (Brilliant SYBR Green PCR Master Mix, Stratagene). The qPCR assays contained 100 nM of each specific forward and reverse primers [40], [41] and 2 µL cDNA as template in a reaction volume of 25 µL. The reactions were performed in the RealPlex real time PCR cycler (Eppendorf). After 10 min denaturation at 95°C, the reactions were cycled 40 times at 95°C for 30 s, 60°C for 1 min and 72°C for 30 s. To verify that only the specific product was amplified, a melting point analysis was done after the last cycle by cooling samples to 55°C followed by increasing the temperature to 95°C at 0.5°C/s. Each run included a non-template control to test for contamination of assay reagents.

### Data analysis

The relative expression ratio of a target gene was calculated based on the PCR efficiency and Ct of the treated sample versus untreated sample using the ΔΔCt method [42]. Glyceraldehyde 3-phosphate dehydrogenase (GAPDH) and β-actin were used as reference genes for normalisation. Data were expressed as mean±SD of three independent experiments. P<0.05 was considered significant compared to the negative control (cells treated with Trx) as analysed by unpaired Student's t-test.

### Parasite and chickens

The Houghton strain of *E. tenella* was maintained by passage through 4–6 week-old specific pathogen free (SPF) White Leghorn chickens. Oocysts were obtained from the caeca of chickens at 7 days post-infection and sporulated according to the previously described method [43]. The SPF chickens were reared in a coccidia-free room established by previous ammonia fumigation and provided food and water ad libitum.

### Preparation of *E. tenella* merozoite crude lysates

The preparation of second-generation merozoites was carried out according to Shirley [43]. SPF White Leghorn chickens were infected with 20,000 sporulated oocysts and, on day 5 post-infection, caeca were removed, cut open longitudinally and divided into smaller pieces. The caeca were then incubated with HBSS containing 10 mM MgCl_2_, 0.25% trypsin and 1% taurocholic acid at 41°C with stirring. The release of merozoites was checked after 10–15 min and generally the maximum number of merozoites appeared after 30 min in the absence of contaminating debris. The merozoites were washed several times by centrifugation at 800× g for 10 min. The merozoites were then freeze-thawed three times and sonicated at 40 Hz with 30 s bursts. The proteins were collected by centrifugation for 15 min at 14,000× g and passed through the polymyxin B agarose column (Detoxi gel, Pierce) and contained less than 0.25 EU/µg protein as determined by the *Limulus* ameabocyte lysate (LAL) assay (QCL-1000 test kit, BioWhittaker).

### Preparation of *E. tenella* crude sporulated oocyst lysates

The sporulated oocyts were suspended in PBS, pH 7.4 and vortexed vigorously with glass beads for 5–10 min. The suspensions were then subjected to a 30 s burst of sonication at 4°C and centrifuged at 14,000× g for 15 min. The supernatants collected were crude sporulated oocyst lysates. The concentration of crude lysates was determined by bicinchoninic acid assay (Sigma).

### Preparation of chicken sera

To examine the immunogenicity of rSAGs, 3 week-old SPF White Leghorn chickens were experimentally infected with a dose of 3,000 *E. tenella* sporulated oocysts. The infected chickens were slaughtered on day 7 (n = 3), day 8 (n = 6) and day 14 (n = 6) post-infection and blood collected for sera preparation [35]. Serum sample were also prepared from six uninfected chickens.

### Enzyme-linked immunosorbent assay (ELISA)

Microtiter plates were coated overnight at 4°C with 1 µg of rSAGs or 5 µg of *E. tenella* sporulated oocyst crude antigens per well in bicarbonate buffer (0.05 NaHCO_3_, pH 9.6). Trx was coated as a negative control at 1 µg per well. Plates were blocked with blocking buffer (5% skim milk in Tris buffered saline (TBS) for 1 hour at 37°C. After washing the plates with TBS, 100 µL of sera (1∶25) were added to each well and incubated at 37°C for 1 h. Plates were then washed with washing buffer (TBS-0.05% Tween 20), and incubated with 100 µL of alkaline phosphatase conjugated goat anti-chicken IgG, IgM or IgA (Bethyl Laboratories) for 1 h at 37°C. The plates were washed with washing buffer and 50 µL of chromogenic substrate 1-step *para*-nitrophenylphosphate (Pierce) was added. The reaction was left to develop for 30 min at room temperature. The absorbance was measured at 405 nm with an automated Sunrise ELISA reader (Tecan). As the rSAGs were expressed as Trx fusion proteins, the absorbance for each rSAG was obtained after subtracting the reading for the control Trx fusion partner (triplicate). The cut off point was determined as the mean of the six uninfected controls plus three standard deviations. A cut off value of 0.2 was then used for all tested rSAGs. For sporulated oocyst antigens, the cut off point was determined as 0.47. A sample was considered positive if the calculated absorbance value was equal to or greater than the cut off point.

## Supporting Information

Figure S1 **SDS-PAGE and western blot analyses of expressed and purified rSAGs.** Expressed and purified rSAGs were analysed by (A) SDS-PAGE and (B) western blot probed with anti-His tag antibody. rSAGs were expressed as soluble Trx fusion proteins in *Escherichia coli* Rosetta gami (DE3) at 20°C following induction with 0.1 mM IPTG for 20 h and further purified under native conditions using immobilised metal affinity chromatography (nickel sepharose). Lanes: 1, expressed crude soluble rSAG; 2, purified rSAG. M, prestained protein marker (New England Biolabs).
(TIF)

Figure S2 **Effect of polymyxin B sulphate on nitrite production by chicken macrophage HTC cells stimulated with** ***Eimeria tenella*** **merozoite crude lysate, rSAGs 4, 5 and 12 and Trx.** Chicken macrophages were exposed to 10 µg/mL of *Eimeria tenella* merozoite crude lysate, rSAGs 4, 5 and 12 and Trx for 24 h with the presence or absence of polymyxin B sulphate (50 µg/mL). LPS (1 µg/mL) was used as a positive control and non-stimulated macrophages (media alone) was used as a negative control. Polymyxin B sulphate did not affect the rSAGs and *E. tenella* merozoite crude lysate-induced nitrite production but significantly abolished that triggered by 1 µg/mL of LPS. The results are expressed as mean±SD of three independent experiments. *, P<0.001 was considered significant as analysed by unpaired Student's t-test.
(TIF)

Table S1 **Primers designed for the amplification of** ***Eimeria tenella*** **SAG transcripts.**
(DOC)

Table S2 **The prevalence of immunopositive reactions against a crude sporulated oocyst antigen lysate (Spo) and each rSAG using sera obtained from chickens infected with a dose of 3,000** ***Eimeria tenella*** **sporulated oocysts on days 8 and 14 post-infection.**
(DOC)
