# Supplementary figures and images for: Immunogenic Eimeria tenella Glycosylphosphatidylinositol-Anchored Surface Antigens (SAGs) Induce Inflammatory Responses in Avian Macrophages
Source: PLoS One. 2011 Sep 28;6(9):e25233. doi: 10.1371/journal.pone.0025233 (PMC3182191; doi:10.1371/journal.pone.0025233)

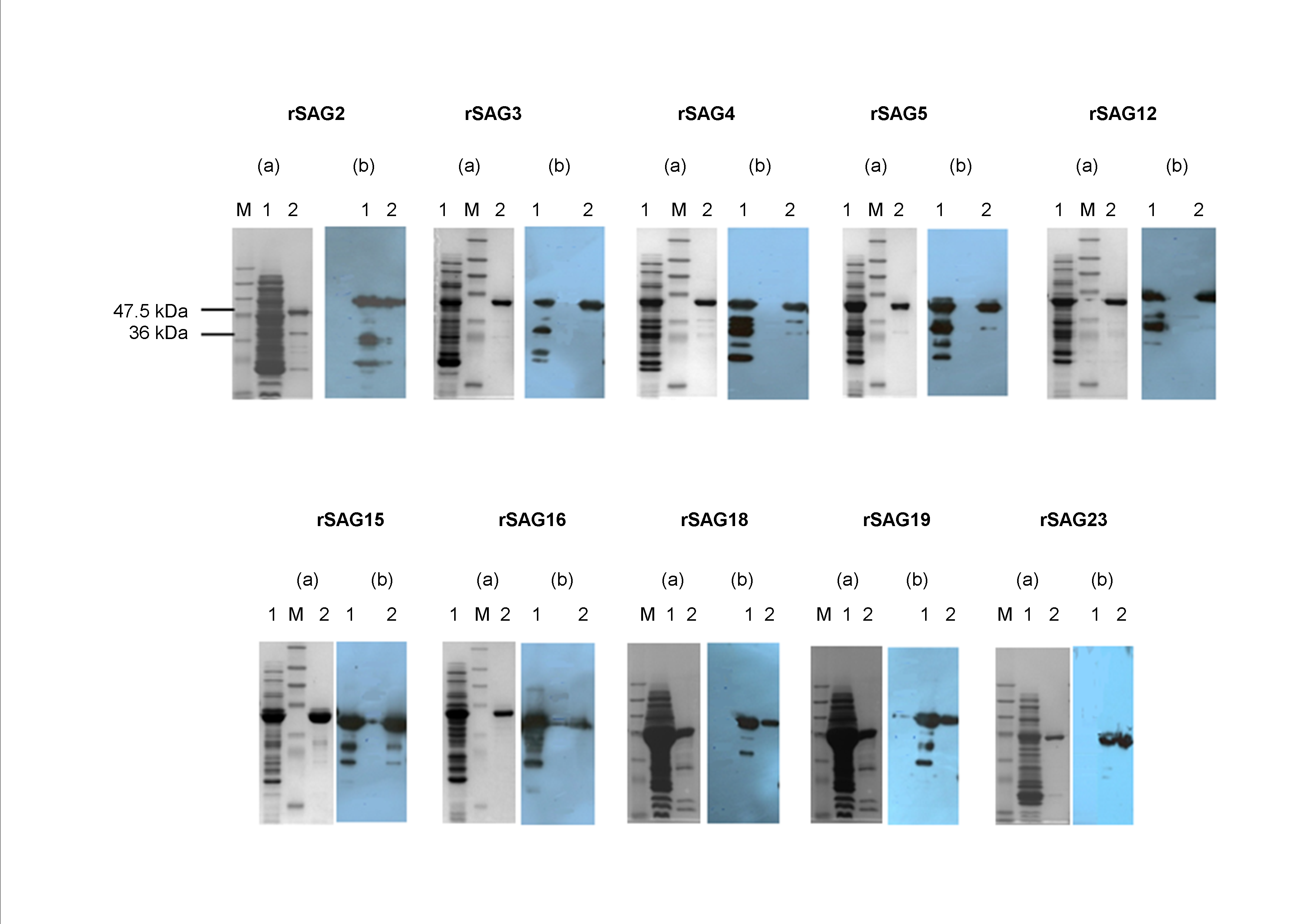

Supplement: Figure S1 — SDS-PAGE and western blot analyses of expressed and purified rSAGs. Expressed and purified rSAGs were analysed by (A) SDS-PAGE and (B) western blot probed with anti-His tag antibody. rSAGs were expressed as soluble Trx fusion proteins in Escherichia coli Rosetta gami (DE3) at 20°C following induction with 0.1 mM IPTG for 20 h and further purified under native conditions using immobilised metal affinity chromatography (nickel sepharose). Lanes: 1, expressed crude soluble rSAG; 2, purified rSAG. M, prestained protein marker (New England Biolabs). (TIF) [file pone.0025233.s001.tif]

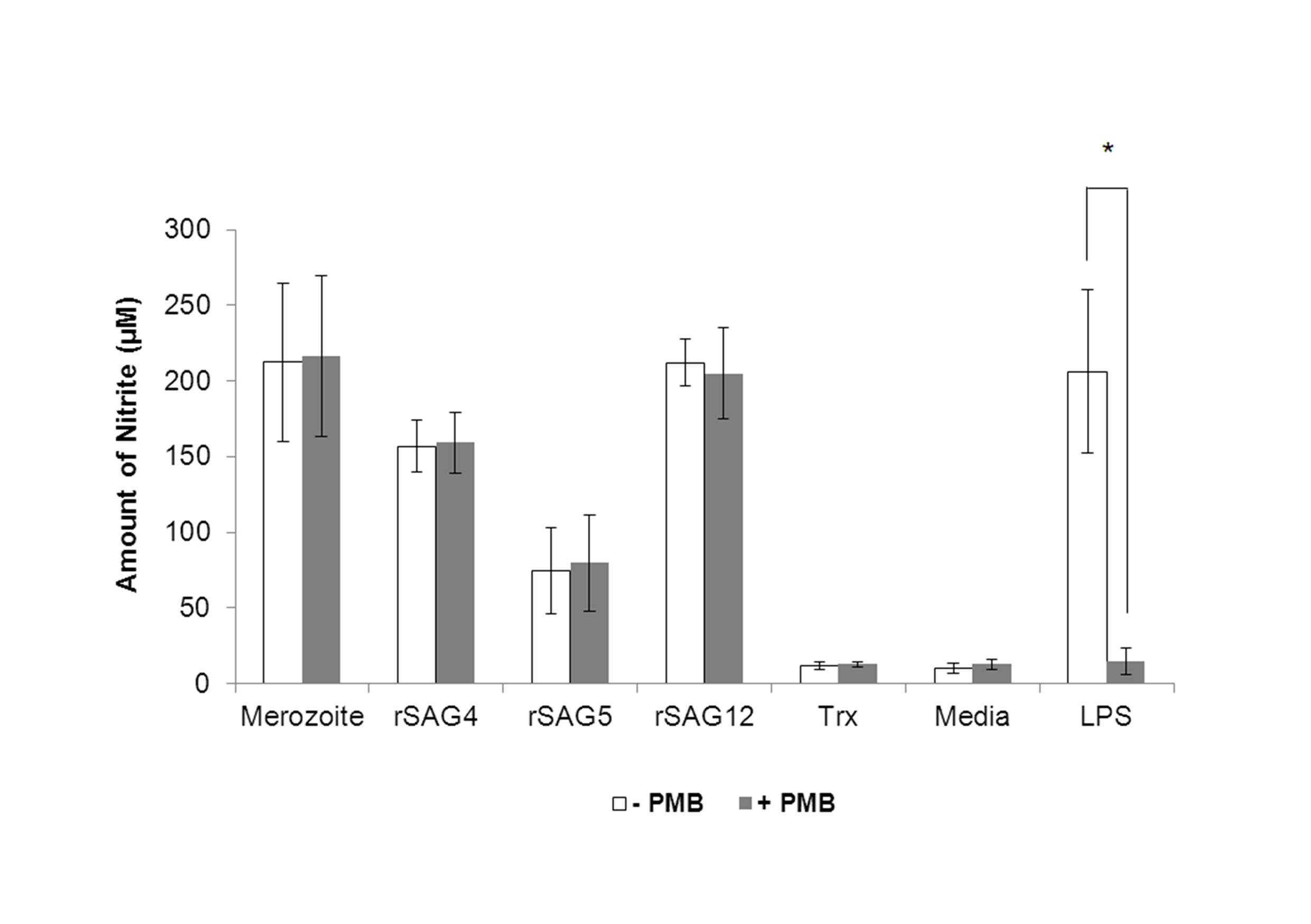

Supplement: Figure S2 — Effect of polymyxin B sulphate on nitrite production by chicken macrophage HTC cells stimulated with Eimeria tenella merozoite crude lysate, rSAGs 4, 5 and 12 and Trx. Chicken macrophages were exposed to 10 µg/mL of Eimeria tenella merozoite crude lysate, rSAGs 4, 5 and 12 and Trx for 24 h with the presence or absence of polymyxin B sulphate (50 µg/mL). LPS (1 µg/mL) was used as a positive control and non-stimulated macrophages (media alone) was used as a negative control. Polymyxin B sulphate did not affect the rSAGs and E. tenella merozoite crude lysate-induced nitrite production but significantly abolished that triggered by 1 µg/mL of LPS. The results are expressed as mean±SD of three independent experiments. *, P<0.001 was considered significant as analysed by unpaired Student's t-test. (TIF) [file pone.0025233.s002.tif]
