# Supplementary material for: Immunogenic Eimeria tenella Glycosylphosphatidylinositol-Anchored Surface Antigens (SAGs) Induce Inflammatory Responses in Avian Macrophages
Source: PLoS One. 2011 Sep 28;6(9):e25233. doi: 10.1371/journal.pone.0025233 (PMC3182191; doi:10.1371/journal.pone.0025233)
Supplement: Table S1 — Primers designed for the amplification of Eimeria tenella SAG transcripts. (DOC) [file pone.0025233.s003.doc]

**Table S1. Primers designed for the amplification of *Eimeria tenella* transcripts.**

| **Transcript** | **Accession no.** | **Primer** | **Transcript size (bp)** | **Protein size**  **(kDa)** |
| --- | --- | --- | --- | --- |
| SAG2 | AJ586540.1 | (F): 5' CCATGGCGAGTGCTGCCGCAACCGA 3' | 710 | ~43 |
|  |  | (R): 5' CTCGAGCCTGACTGGCGAAACCCCGT 3' |  |  |
| SAG3 | AJ586553.1 | (F): 5' CCATGGCGGGCACAACGGGAACAG 3' | 660 | ~42 |
|  |  | (R): 5' CTCGAGCCTGACTGGCGAAATCCCG 3' |  |  |
| SAG4 | AJ586535.1 | (F): 5' CCATGGCGCAGCAACAAGCTGCTACTCC 3' | 660 | ~41 |
|  |  | (R): 5' CTCGAGGCCCACTGGGGAAACTTCGCT 3' |  |  |
| SAG5 | AJ586532.1 | (F): 5' CCATGGCGTTGGGCAGCTCACAAG 3' | 650 | ~41 |
|  |  | (R): 5' CTCGAGCGTGAGCGAAGATATGATTCTGTC 3' |  |  |
| SAG12 | AJ586534.1 | (F): 5' CCATGGCGGATGGGACGCCTGAGG 3' | 660 | ~41 |
|  |  | (R): 5' CTCGAGCGAGCCTGTGAGAGATGATACGATT 3' |  |  |
| SAG15 | AJ586550.1 | (F): 5' CCATGGCGGCCACATCCAATTTCTCAGT 3' | 690 | ~42 |
|  |  | (R): 5' CTCGAGACCGGCCTGCCGCTGTAAGA 3' |  |  |
| SAG16 | AJ586542.1 | (F): 5' CCATGGCGGGTGCAATCATCACTCGCTCTG 3' | 680 | ~42 |
|  |  | (R): 5' CTCGAGACCTGCCTGCCGCTGCAAGAGAGC 3' |  |  |
| SAG18 | AJ586548.1 | (F): 5' CCATGGCGTTGGCGCTTTCCCTTCGTTCTA 3' | 700 | ~43 |
|  |  | (R): 5' CTCGAGGGGAACTGATGTTGACGCTGGGC 3' |  |  |
| SAG19 | AJ586544.1 | (F): 5' CCATGGCGGCCGCACCAGACTTCTC 3' | 710 | ~43 |
|  |  | (R): 5' CTCGAGTGCTTCCAATCCCCACAGAGCATT 3' |  |  |
| SAG23 | AJ586547.1 | (F): 5' CCATGGCGACCGCCGCTCCACACT 3' | 700 | ~43 |
|  |  | (R): 5' CTCGAGCAATCCCCAGAGGGCATTGTACAG 3' |  |  |

(F) = forward primer, (R) = reverse primer.
