# Supplementary material for: Immunogenic Eimeria tenella Glycosylphosphatidylinositol-Anchored Surface Antigens (SAGs) Induce Inflammatory Responses in Avian Macrophages
Source: PLoS One. 2011 Sep 28;6(9):e25233. doi: 10.1371/journal.pone.0025233 (PMC3182191; doi:10.1371/journal.pone.0025233)
Supplement: Table S2 — The prevalence of immunopositive reactions against a crude sporulated oocyst antigen lysate (Spo) and each rSAG using sera obtained from chickens infected with a dose of 3,000 Eimeria tenella sporulated oocysts on days 8 and 14 post-infection. (DOC) [file pone.0025233.s004.doc]

**Table S2.** The prevalence of immunopositive reactions against a crude sporulated oocyst antigen lysate (Spo) and each rSAG using sera obtained from chickens infected with a dose of 3,000 *Eimeria tenella* sporulated oocysts on days 8 and 14 post-infection.

| **Antigen** | **No. of positively reactive chickens (%)** | |
| --- | --- | --- |
|  | **Day 8 (n=6)** | **Day 14 (n=6)** |
| Spo | 5/6 (83) | 3/6 (50) |
| rSAG2 | 4/6 (67) | 6/6 (100) |
| rSAG3 | 6/6 (100) | 6/6 (100) |
| rSAG4 | 5/6 (83) | 6/6 (100) |
| rSAG5 | 2/6 (33) | 4/6 (67) |
| rSAG12 | 6/6 (100) | 6/6 (100) |
| rSAG15 | 5/6 (83) | 6/6 (100) |
| rSAG16 | 6/6 (100) | 6/6 (100) |
| rSAG18 | 2/6 (33) | 5/6 (83) |
| rSAG19 | 5/6 (83) | 6/6 (100) |
| rSAG23 | 4/6 (67) | 5/6 (83) |
